# ETS2 targets ZMYND11 to inhibit thyroid cancer progression via the mTOR signaling pathway

**DOI:** 10.1371/journal.pone.0328881

**Published:** 2025-09-12

**Authors:** Taipengfei Shu, Xinhua Wu, Chengqun Wei, Chaofeng Chen, Chao Shen, Yujie Huang, Jie Zhou, Liangxing Jiang, Ting Yan, Wen Shi, Liming Ma, Yan Yan, Tao Yu, Ning Ji, Jun Jiang, Xiangyu Xie, Ping Zhu

**Affiliations:** 1 Department of Endocrinology, Huai’an Clinical Medical College of Jiangsu University & Huai’an Hospital of Huai’an City & Huai’an Cancer Hospital &The Affiliated Huai’an Hospital of Jiangsu College Of Nursing, Huai’an City, Jiangsu Province, China; 2 Jiangsu Key Laboratory of Medical Science and Laboratory Medicine, School of Medicine, Jiangsu university, Zhenjiang, Jiangsu, P.R. China; 3 Department of Central Laboratory, Huai'an Clinical Medical College of Jiangsu University & Huai'an Hospital of Huai'an City & Huai'an Cancer Hospital &The Affiliated Huai'an Hospital of Jiangsu College Of Nursing, Huai'an City, Jiangsu Province, China; 4 Department of Neurology, Huai’an Clinical Medical College of Jiangsu University & Huai’an Hospital of Huai’an City & Huai’an Cancer Hospital &The Affiliated Huai’an Hospital of Jiangsu College Of Nursing, Huai’an City, Jiangsu Province, China; 5 Department of cardiovascular medicine, Huai’an Clinical Medical College of Jiangsu University & Huai’an Hospital of Huai’an City & Huai’an Cancer Hospital &The Affiliated Huai’an Hospital of Jiangsu College Of Nursing, Huai’an City, Jiangsu Province, China; 6 Department of Clinical Laboratory, Huai’an Clinical Medical College of Jiangsu University & Huai’an Hospital of Huai’an City & Huai’an Cancer Hospital &The Affiliated Huai’an Hospital of Jiangsu College of Nursing, Huai’an, China; 7 Endoscopy Center, Minhang Hospital, Fudan University, Shanghai, China,; 8 Department of General Surgery, Huai’an Clinical Medical College of Jiangsu University & Huai’an Hospital of Huai’an City & Huai’an Cancer Hospital & The Affiliated Huai’an Hospital of Jiangsu College of Nursing, Huai’an City, Jiangsu Province, China,; 9 Department of Endocrinology, The Affiliated Chuzhou Hospital of Traditional Chinese Medicine of Jiangsu College of Nursing, Huai’an, China; Xiangya Hospital Central South University, CHINA

## Abstract

**Background:**

Despite advancements in thyroid cancer (THCA) treatment, the prognosis for advanced cases remains poor. Cellular senescence is crucial in tumor progression, with *ETS2* emerging as a key regulator. However, the role of *ETS2* and its interaction with *ZMYND11* in THCA is unclear.

**Methods:**

Differentially expressed genes (DEGs) connected with cellular senescence were determined from The Cancer Genome Atlas (TCGA)-THCA dataset. Functional analysis, prognostic risk model, and nomogram were then performed to identify *ETS2* as a hub gene. The roles of *ETS2* and *ZMYND11* were explored using Western blotting (WB), co-immunoprecipitation (Co-IP), and quantitative real-time polymerase chain reaction (qRT-PCR). Effects of *ETS2* overexpression and knockdown of *ZMYND11* on apoptosis, cell proliferation, epithelial-mesenchymal transition (EMT), and mTOR signaling were evaluated. *In vivo*, a xenograft model was established using Cal-62 cells with or without *ETS2* overexpression to assess tumor growth and protein expression.

**Results:**

*ETS2* was notably downregulated in THCA, and its low expression was connected to adverse prognosis. *ETS2* overexpression inhibited THCA cell invasion, migration, proliferation, and induced apoptosis. *ETS2* also regulated the expression of EMT markers, indicating its role in inhibiting THCA progression. Co-IP analysis showed that ETS2 interacted with ZMYND11. Knockdown of *ZMYND11* attenuated the inhibitory effect of *ETS2* on THCA cell behavior and mTOR pathway regulation. *In vivo*, *ETS2* overexpression reduced tumor growth and increased *ETS2* and *ZMYND11* expression in xenograft tumors.

**Conclusion:**

This study identified the cellular senescence gene *ETS2* as a tumor suppressor in THCA, which interacts with *ZMYND11* to regulate THCA tumor progression through the mTOR pathway, thereby inhibiting cell senescence. Targeting the *ETS2*-*ZMYND1* axis may provide new therapeutic strategies and prognostic biomarkers for THCA.

## Introduction

As a potentially lethal malignant tumor, thyroid cancer (THCA) grows rapidly [1]. It is highly invasive, especially in the undifferentiated form, with a negative prognosis and low survival rate, presenting a serious threat to the health of patients. Papillary thyroid cancer accounts for 80–90% of occurrences of thyroid cancer, making it the most prevalent kind of all types [2]. Since 2000, the occurrence of THCA has increased by 20% every year, and by 2022 it has become the third most prevalent cancer in China [3]. THCA is more prevalent in females than in males and is specifically prevalent in young and elderly people [4]. Current treatment strategies for thyroid cancer include radioactive iodine therapy, surgical intervention, targeted therapy, and immunotherapy [5]. Cellular senescence refers to a stable state of proliferation arrest in cells after irreversible damage or stress. Recently, the function of cell senescence in THCA has received increasing attention. The senescence-associated secretory phenotype (SASP) may serve a promoting function in tumor growth and metastasis by promoting angiogenesis, tumor cell proliferation, and immune escape [6]. Studies have shown that senescence-related characteristics may become important biomarkers for estimating the prognosis of THCA and guiding immunotherapy, such as *DOCK6* and *ADAMTSL4* [7]. These findings offer fresh perspectives on the diagnosis and management of thyroid cancer and emphasize the significance of aging mechanisms in cancer research.

*ETS2* is a transcription factor closely related to cell senescence and belongs to the ETS family. It is essential for many biological activities, comprising cell proliferation, apoptosis, differentiation, development, and tumorigenesis [8]. As one of the cell senescence-related genes, *ETS2* is involved in regulating multiple key cell signaling pathways in tumors, such as the JAK/STAT, RAS/MAPK, and PI3K/AKT pathways, which perform an essential function in the occurrence and progression of cancer. In some cancer types, *ETS2* exhibits oncogenic properties. For example, overexpression of *ETS2* is intimately related to tumor progression, invasion, and metastasis in breast cancer, colorectal cancer, and prostate cancer [9]. Research findings have also indicated that *ETS2* may encourage the development of cancer by activating genes like Myc and Cyclin D1 that are involved in cell survival and proliferation [10]. Additionally, *ETS2* has the potential to act as a specific marker for the early detection of acute myocardial infarction (AMI), and its early detection capability provides a new opportunity for timely intervention in cardiovascular diseases [11]. Cellular senescence-related gene markers have also been shown to reliably predict patient prognosis and immunotherapy efficacy, especially in immunotherapy for hepatocellular carcinoma, in which *ETS2* is a key research subject.

*ZMYND11*, also known as *BS69*, is a zinc finger domain-containing protein belonging to the MYND domain protein family. *ZMYND11* can function as a co-repressor of transcription, interacting with promoter regions of specific genes and inhibiting their transcription [12]. Previous studies have indicated interactions between *ETS2* and *ZMYND11*. Research by Plotnik JP et al. demonstrated that *ETS1* and *ETS2* have opposing regulatory roles in the gene expression program for cell migration, with the function of ETS2 being specifically modulated by the co-repressor *ZMYND11*, which determines its oncogenic or tumor-suppressive roles in different cellular contexts [13]. Similarly, Wei G et al. found that *BS69* acts as a co-repressor interacting with *ETS2*, and phosphorylation of *ETS2* reduces this interaction, potentially shifting its function from a repressor to an activator [14]. Therefore, this research aims to investigate the roles and mechanistic pathways of *ETS2* and *ZMYND11* in THCA, thereby providing new directions for the treatment of THCA.

Despite advances in THCA treatment, including radioiodine therapy, surgery, targeted therapy, and immunotherapy, the prognosis of advanced cases remains poor. Recent studies have highlighted key factors in the progression of THCA, like *ETS2*, a transcriptional regulator implicated in tumor development and metastasis in various cancers [15]. *ETS2* is known to regulate key cellular processes by modulating multiple signaling pathways. This research attempts to clarify the function of *ETS2* in THCA by exploring its interaction with *ZMYND11*. By investigating their effects on cell proliferation, epithelial-mesenchymal transition (EMT), apoptosis, and the mTOR pathway, this investigation aims to find possible therapeutic targets and biomarkers to ultimately improve the prognosis and therapeutic strategies of THCA.

## Materials and methods

### Analysis of differentially expressed genes related to cellular senescence in THCA

Samples connected to THCA were acquired from The Cancer Genome Atlas (TCGA) database via the Sangerbox tool (http://vip.sangerbox.com/home.html) for this research. The dataset included 512 THCA tumor samples and 59 adjacent normal samples from TCGA. The selection of cutoff values for DEGs was based on commonly accepted thresholds in bioinformatics studies to balance the sensitivity and specificity of gene identification. DEGs were characterized based on a log_2_ fold change (FC) threshold of <−1 for downregulated genes and >1 for upregulated genes, as this level of change is generally considered significant in gene expression studies. Additionally, a P-value threshold of <0.05 was applied to ensure the statistical robustness of the identified DEGs, reducing the likelihood of false positives. These criteria were selected to provide a reliable and interpretable set of DEGs for subsequent analyses. To investigate the relationship between DEGs and cellular senescence, 278 genes connected with cell senescence were acquired from the CellAge database (https://genomics.senescence.info/cells/). Intersection analysis of cell senescence-related genes and DEGs was performed using the Venn online graph tool (https://bioinformatics.psb.ugent.be/webtools/Venn/) to identify overlapping genes.

### Gene Ontology (GO) and Kyoto Encyclopedia of Genes and Genomes (KEGG) pathway enrichment analysis

The enrichment analysis for cellular senescence-associated genes in THCA, focusing on GO and KEGG pathways, was conducted via the Xiantao Academic platform (https://www.xiantao.love/products). This analysis sought to highlight key biological processes, cellular structures, molecular functions, and signaling pathways. A *P*-value < 0.05 was applied as the significance threshold for all results obtained from the enrichment analysis.

### Univariate Cox regression analysis and protein-protein interaction (PPI) analysis

To evaluate the prognostic significance of cellular senescence-related genes in THCA, we employed univariate Cox regression analysis. Cox regression is based on the proportional hazards assumption, which posits that the hazard ratio for the effect of a covariate is constant over time. To ensure the validity of this assumption in our analysis, we evaluated the proportional hazards through diagnostic tests. 95% confidence intervals (CIs) and Hazard ratios (HRs) were measured to determine genes significantly connected to overall survival. *P*-values < 0.05 suggested that the findings were statistically significant. In addition, with the Search tool for the retrieval of interacting genes/proteins (STRING; https://string-db.org/) database, PPI networks were built, and Cytoscape software (version 3.10.2) was utilized to display the results.

### Prognostic feature construction and risk model evaluation using least absolute shrinkage and selection operator (LASSO) regression

The creation of prognostic characteristics used the LASSO regression analysis in R, utilizing the “glmnet” package. LASSO regression assumes that there is sparsity among the features, meaning that only a small subset of the variables were truly associated with the outcome, while others have coefficients shrunk to zero. This assumption ensures that the model can effectively handle high-dimensional data and prevent overfitting. LASSO regression was employed to identify relevant prognostic features from a set of nine prognostic genes, with optimal gene selection determined through 10-fold cross-validation. Based on this analysis, the following algorithm was used to determine a risk score for every patient: Riskscore=(0.2922)*MMP9+(0.7243)*SRSF1+(−0.4812)*FASTK+(−0.1894)*CBX7+(−1.0796)*ETS2. Based on the median value of the computed risk scores, groups of patients then categorized as high-risk and low-risk were formed. Kaplan-Meier survival curves were generated to compare survival differences between these groups utilizing the “survival” package in R. The “timeROC” R package was utilized to create receiver operating characteristic (ROC) curves, which evaluated the predictive capacity of the risk model. Throughout the studies, statistical significance was measured with a threshold of *P* < 0.05.

### Construction of prognostic nomogram for THCA

Five key signature genes were identified from the prognostic risk model. To evaluate the relevance of the prognosis of these selected genes and clinical variables (comprising age, gender, pN stage, pT stage, and pM stage), univariate and multivariate Cox regression analyses were performed via the “survival” R package. To find independent prognostic factors, variables having a *P*-value < 0.05 in the univariate analysis were added to the multivariate model. Subsequently, the “rms” R program was employed to create a nomogram that predicted the overall survival rates at 1, 3, and 5 years. Calibration curves were generated to evaluate the concordance between observed and predicted survival rates, enabling the identification of hub genes in this study.

### Expression analysis and survival analysis of ETS2 in pan-cancer

To validate the biological characteristics of the hub gene, we initially conducted a pan-cancer expression analysis, focusing particularly on THCA tumor tissues, utilizing the Gene Expression Profiling Interactive Analysis (GEPIA) database (http://gepia.cancer-pku.cn/). This platform provides comprehensive gene expression profiling among many forms of cancer, allowing for the assessment of differential expression patterns. Subsequently, Kaplan-Meier survival analyses were performed to assess the impact of differential expression of these key genes on progression-free survival (PFS) and disease-free survival (DFS) in THCA patients, determining the prognostic significance associated with their expression levels.

### Immunohistochemical (IHC) analysis of ETS2 in THCA tissues

Thyroid tissue samples that were formalin-fixed, paraffin-embedded (FFPE) were provided by the Shanghai Minhang Central Hospital on 28/06/2024 and authorized by the Institutional Ethics Committee (2024-Approval-015–112). IHC staining was performed using an IHC kit (BOSTER, China). After being deparaffinized and rehydrated, the tissue slices were incubated at 95°C for ten minutes in Tris-EDTA buffer (Solarbio, China) for antigen retrieval. An incubation period of 10 minutes at room temperature inhibited endogenous peroxidase activity. After blocking the sections with 5% bovine serum albumin (BSA) for 30 minutes at room temperature, they were treated with an anti-ETS2 antibody (1:100, Abcam, China) overnight at 4°C. Following incubation, the slices were subjected to a biotin-labeled goat anti-mouse IgG secondary antibody and visualized using a DAB substrate kit (ZSGB, Beijing, China). subsequently, 1% hematoxylin was applied to the slices for counterstaining and imaged using a Leica DMI3000B microscope.

### Ethics approval and consent to participate

Our study was conducted with the approval of the Ethics Committee of Shanghai Minhang Central Hospital, and informed consent for the relevant research was obtained(2024-approval-015-112).

All animal experiments were performed in accordance with the guidelines for the care and use of laboratory animals and were approved by the Animal Ethics Committee of Hefei National Comprehensive Science Center Institute of Health (Approval No. IHM-API-2025-007). All efforts were made to minimize animal suffering and reduce the number of animals used.

### Cell culture conditions

THCA cell lines (Cal-62, TPC-1, and BC-PAP) and normal thyroid epithelial cells (Nthy-ori 3-1) were acquired from Procell (Wuhan, China). Every cell was cultured in RPMI-1640 medium (Gibco, Shanghai, China), which was enhanced with 1% penicillin-streptomycin and 10% fetal bovine serum (FBS). At 37°C, the cells were maintained in a humidified atmosphere with 5% CO_2_.

### Cell transfection

In 24-well plates, Cal-62 and TPC-1 cells were seeded at a density of 2 × 10^4^ cells per well and grown for a whole night in a complete growth medium to reach approximately 70–80% confluence. By the manufacturer’s instructions, Lipofectamine 2000 (BioSharp, China) was employed for transfections. ETS2 overexpression plasmids and *ZMYND11* knockdown plasmids, along with their respective negative controls (over-NC, si-NC), were introduced into the cells. The sequence used for the *ZMYND11* knockdown was GAAGGGAAATACCGAAGTTAT. 48 hours after transfection, cells were collected for further analyses. All transfection procedures followed standardized protocols to ensure reproducibility and accuracy.

### Quantitative real-time polymerase chain reaction (qRT-PCR)

TRIzol reagent (Thermo Fisher Scientific, Shanghai, China) was applied to extract total RNA from Cal-62 and TPC-1 cells by the manufacturer’s instructions. The quantity and quality of RNA were assessed utilizing a NanoDrop spectrophotometer (Thermo Fisher Scientific, Shanghai, China). Subsequently, cDNA synthesis was performed via the PrimeScript RT Kit (Takara, Shiga, Japan) with 1 μg of total RNA as the template. To analyze the gene expression levels, qRT-PCR was conducted by the StepOnePlus Real-Time PCR System (Applied Biosystems, Shanghai, China). SYBR Green PCR Master Mix (Applied Biosystems, Shanghai, China) was utilized for qRT-PCR reactions. Gene expression analysis was conducted utilizing the 2^-ΔΔCT^ method, with β-actin serving as the internal control for normalization. The primer sequences employed in qRT-PCR are listed in Table 2.

**Table 2:** Primer sequences for qRT-PCR.

Target	Direction	Sequence (5’-3’)
*ETS2*	Forward	GGCCCGGTTTCTACAGGAAG
*ETS2*	Reverse	CTTTGGAATTCCGCAGCGAC
*ZMYND11*	Forward	ACAAAAAGACGACAGGCGGA
*ZMYND11*	Reverse	ACGGGTGGTCTCTTTAGGGT
*Bax*	Forward	CTGCAGAGGATGATTGCCG
*Bax*	Reverse	TGCCACTCGGAAAAAGACCT
*Bcl-2*	Forward	TCCCTCGCTGCACAAATACTC
*Bcl-2*	Reverse	ACGACCCGATGGCCATAGA
*Caspase-3*	Forward	AGGGGTCATTTATGGGACA
*Caspase-3*	Reverse	TACACGGGATCTGTTTCTTTG
*Caspase-9*	Forward	AGGCCCCATATGATCGAGGA
*Caspase-9*	Reverse	TCGACAACTTTGCTGCTTGC
*E-cadherin*	Forward	ACATACACTCTCTTCTCTC
*E-cadherin*	Reverse	GTCATTCTGATCGGTTAC
*N-cadherin*	Forward	GGGTGGAGGAGAAGAAGACCAG
*N-cadherin*	Reverse	GGCATCAGGCTCCACAGT
*Vimentin*	Forward	AACCTGAGGGAAACTAAT
*Vimentin*	Reverse	TTGATAACCTGTCCATCT
*ZEB1*	Forward	GAAAGTGTTACAGATGCAG
*ZEB1*	Reverse	TTCCTTTCCTGTGTCATC
*ZEB2*	Forward	ATGAAGCAGCCGATCATGGCG
*ZEB2*	Reverse	CACACATCTTGGAGCAAAAGCATG
*Snail*	Forward	GAAAAGGGACTGTGAGTA
*Snail*	Reverse	GAATAGTTCTGGGAGACA
*Slug*	Forward	CTGGTCAAGAAGCATTTC
*Slug*	Reverse	GGGGAAATAATCACTGTATG
*S6K1.*	Forward	CACATAACCTGTGGTCTGTTGCTG
*S6K1.*	Reverse	AGATGCAAAGCGAACTTGGGA
*NF-κB1*	Forward	GCCTCCACAAGGCAGCAAATA
*NF-κB1*	Reverse	CACCACTGGTCAGAGACTCGGTAA
*COX2*	Forward	AAGTCC CTGAGCATCTACG
*COX2*	Reverse	TTCCTA CACCAGCAACC
*4EBP1*	Forward	TATGACCGGAAATTCCTGATG
*4EBP1*	Reverse	CCATCTCAAACTGTGACTCTTCA
*β-actin*	Forward	TCCTTCCTGGGCATGGAG
*β-actin*	Reverse	AGGAGGGGCAATGATCTT

### Western blotting (WB)

By applying RIPA lysis buffer that has been enhanced with inhibitors (Thermo Fisher Scientific, Shanghai, China) of proteases and phosphatases, protein lysates of Cal-62 and TPC-1 cells were generated. With a BCA protein assay kit (Beyotime, Jiangsu, China), the protein content was ascertained. Proteins in equal quantities were separated by SDS-PAGE and then put onto a PVDF membrane (BSA, Beyotime, Jiangsu, China). All antibodies were purchased from Abcam, Shanghai, China. Primary antibodies were employed to identify the following proteins on the membrane: ETS2, Bax, Caspase-3, Bcl-2, Caspase-9, N-cadherin, E-cadherin, Vimentin, ZEB1, ZEB2, Snail, Slug, ZMYND11, S6K1, NF-κB, COX2, and 4EBP1, with β-actin serving as a loading control. Except for 4EBP1 and β-actin, which were diluted at 1:2000, all other antibodies were diluted at 1:1000. An ECL chemiluminescence detection kit (Beyotime, Jiangsu, China) was applied to observe the protein bands, following incubation with secondary antibodies. A densitometric analysis was executed via the ImageJ software (version 2.0).

### Cell Counting Kit-8 (CCK-8) assay

The CCK-8 test (CK04, Dojindo, China) was employed to assess the activity of cell proliferation. After treatment, Cal-62 and TPC-1 cells were cultured at a concentration of 5 × 10^3^ cells per well in 96-well plates. Ten microliters of CCK-8 reagent were supplied to every well at the predetermined intervals of time (0, 24, 48, 72, and 96 hours). The plates were then cultured for 4 hours at 5% CO_2_ at 37°C in a cell culture incubator. Following the incubation period, a Thermo Fisher Scientific microplate reader (Shanghai, China) was employed to determine the 450 nm absorbance.

### Transwell migration and invasion assay

Cells were harvested and resuspended at a concentration of 5 × 10^4^ cells/well 24 hours after the experiment. These cells were then added into the top chamber of a Transwell insert (24-well format). To replicate the extracellular matrix barrier, Matrigel (Corning) was pre-coated on the top chamber for the invasion experiment. The lower chamber was supplemented with a complete medium to act as a chemoattractant. Following 48 hours of incubation at 37°C, cells remaining in the upper chamber, which did not migrate or invade, were carefully eliminated with a cotton swab. situated beneath the membrane were adhered to utilizing 4% paraformaldehyde and stained with DAPI to visualize the nuclei. Migrated or invaded cells were then visualized and quantified under a fluorescence microscope. Images were subsequently captured for documentation.

### Flow cytometry

To evaluate cell apoptosis in TPC-1 and Cal-62 cells, flow cytometry analysis was performed in this work. After being plated on 24-well plates at a density of 1 × 10^4^ cells per well, the Cal-62 and TPC-1 cells were cultured for 24 hours. Following dissociation with trypsin-EDTA and washing with PBS, cells were cultured with 5 μL of PI and 5 μL Annexin V-FITC of solution at ambient temperature for 15 minutes for apoptosis analysis. Subsequently, a flow cytometer (Jiyuan, Guangzhou, China) was used to assess the staining of PI and Annexin V, and FlowJo software was used to analyze the results.

### Co-immunoprecipitation (Co-IP) assays

Co-IP assays were conducted to validate the relationship between ETS2 and ZMYND11. Plasmids with overexpressed ETS2 or empty vector controls were transfected into Cal-62 and TPC-1 cells. Forty-eight hours post-transfection, protease inhibitor-containing IP lysis solution (Thermo Fisher Scientific, USA) was employed to lyse the cells. The lysates were incubated with an anti-ETS2 antibody for a whole night at 4°C with gentle rotation to allow for immune complex formation. These complexes were then captured using Protein A/G agarose beads (Thermo Fisher Scientific, USA), followed by washing three times with cold lysis buffer to remove non-specifically bound proteins. The bound proteins were dissociated by heating the beads in the SDS-PAGE loading buffer.

### Mouse xenograft model

Six BALB/c nude mice, aged 4–5 weeks, were obtained from Vital River Laboratory Animal Technology Co., Ltd. The mice were housed in specific pathogen-free (SPF) conditions with a 12-hour light/dark cycle and were provided with food and water ad libitum. All animal experiments were approved by the Hefei National Comprehensive Science Center Institute of Health (Approval No.: IHM-API-2025–007) and were conducted in accordance with the guidelines for the care and use of laboratory animals.

The mice were randomly divided into two groups (n = 3 per group). Cal-62 thyroid cancer cells were transfected with an expression plasmid (over-*ETS2* group) or an empty vector (vector) and suspended in PBS. Each mouse was subcutaneously injected with 1 × 10⁶ cells into the right flank. All mice were euthanized on day 24 after injection. Tumors were carefully excised, photographed, and weighed. The tumor tissues were fixed in 4% paraformaldehyde, embedded in paraffin, and sectioned for subsequent IHC analysis of ETS2 and ZMYND 11 expression.

The IHC experiments were performed as described previously. The antibodies used were anti-ETS2 (1:100, Abcam, China) and anti-ZMYND11 (1:250, Abcam, China). Imaging was conducted using a Leica DMI3000B microscope.

### Statistical analysis

For statistical analysis of our dataset, the R programming language was employed. Student’s t-test was utilized to assess intergroup differences and mean values along with their standard deviations (SD) were reported. Tukey’s post-hoc test was performed in conjunction with analysis of variance (ANOVA) to evaluate differences between several groups. There was a predefined significance level of *P* < 0.05.

## Results

### Identification and functional analysis of cellular senescence-related genes in THCA

**Fig 1:**
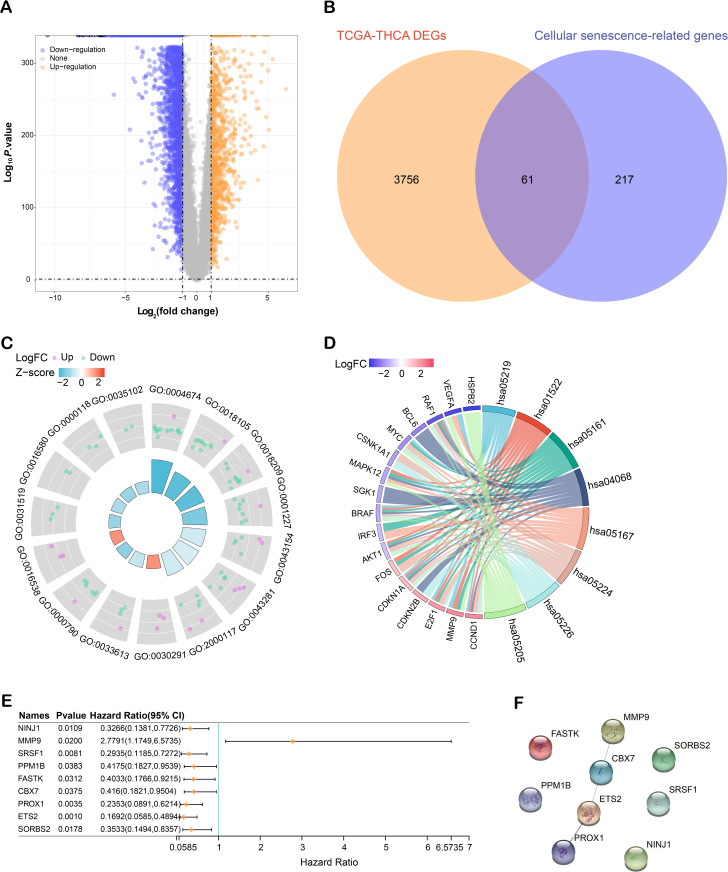
Analysis of cellular senescence-related genes in THCA. **(A)** Volcano plot illustrating the distribution of DEGs in the TCGA-THCA dataset. Upregulated genes are indicated in orange and downregulated genes in blue. **(B)** Venn diagram showing the intersection of DEGs with genes associated with cellular senescence, highlighting overlapping genes. **(C)** GO enrichment analysis of DEGs associated with cellular senescence in THCA. The outer ring represents individual GO terms and their associated DEGs, color-coded by log fold change (Log FC), with upregulated genes in pink and downregulated genes in green. The inner heatmap ring visualizes the Z-score corresponding to each GO term, indicating the overall enrichment trend from downregulated (blue) to upregulated (red) terms. **(D)** KEGG pathway analysis of the 61 overlapping genes, depicting the major pathways they are involved in. The connections between pathways (hsa codes) and genes are color-coded based on LogFC, and the intensity represents the magnitude of differential expression. **(E)** Forest plot from univariate Cox regression analysis showing hazard ratios (HR) and 95% confidence intervals (CI) for nine key genes related to prognosis. **(F)** Protein-protein interaction (PPI) network of nine prognostic genes, with nodes representing genes and edges representing interactions. DEGs: Differentially Expressed Genes; TCGA: The Cancer Genome Atlas; GO: Gene Ontology; KEGG: Kyoto Encyclopedia of Genes and Genomes.

This research identified DEGs from the TCGA-THCA dataset. Following criteria based on FC and *P*-value, we detected 3,260 downregulated and 557 upregulated DEGs (Fig 1A). A topological analysis was conducted further to understand the connection between these DEGs and cellular senescence, revealing 61 overlapping genes (Fig 1B). After that, GO and KEGG enrichment analyses were employed on these 61 overlapping genes to elucidate their functional roles and contributions to biological processes (Figs 1C, 1D; detailed results in Table 1). Univariate Cox regression analysis was applied to refine these genes, identifying nine crucial genes associated with prognosis (Fig 1E). To gain insights into the complex intracellular processes and signaling pathways, a PPI network analysis was performed on the nine prognostic genes (Fig 1F).

**Table 1:** GO and KEGG pathway enrichment analysis of differentially expressed genes in THCA.

ONTOLOGY	ID	Description
BP	GO:0018105	peptidyl-serine phosphorylation
BP	GO:0018209	peptidyl-serine modification
BP	GO:0043154	negative regulation of cysteine-type endopeptidase activity involved in apoptotic process
BP	GO:0043281	regulation of cysteine-type endopeptidase activity involved in apoptotic process
BP	GO:2000117	negative regulation of cysteine-type endopeptidase activity
CC	GO:0000790	nuclear chromatin
CC	GO:0031519	PcG protein complex
CC	GO:0016580	Sin3 complex
CC	GO:0000118	histone deacetylase complex
CC	GO:0035102	PRC1 complex
MF	GO:0004674	protein serine/threonine kinase activity
MF	GO:0001227	DNA-binding transcription repressor activity, RNA polymerase II-specific
MF	GO:0030291	protein serine/threonine kinase inhibitor activity
MF	GO:0033613	activating transcription factor binding
MF	GO:0016538	cyclin-dependent protein serine/threonine kinase regulator activity
KEGG	hsa05219	Bladder cancer
KEGG	hsa01522	Endocrine resistance
KEGG	hsa05161	Hepatitis B
KEGG	hsa04068	FoxO signaling pathway
KEGG	hsa05167	Kaposi sarcoma-associated herpesvirus infection
KEGG	hsa05224	Breast cancer
KEGG	hsa05226	Gastric cancer
KEGG	hsa05205	Proteoglycans in cancer

GO, gene ontology; BP, biological process; CC, cellular component; MF, molecular function; KEGG, kyoto encyclopedia of genes and genomes.

### Identification of prognostic genes for THCA using LASSO regression and risk model evaluation

**Fig 2:**
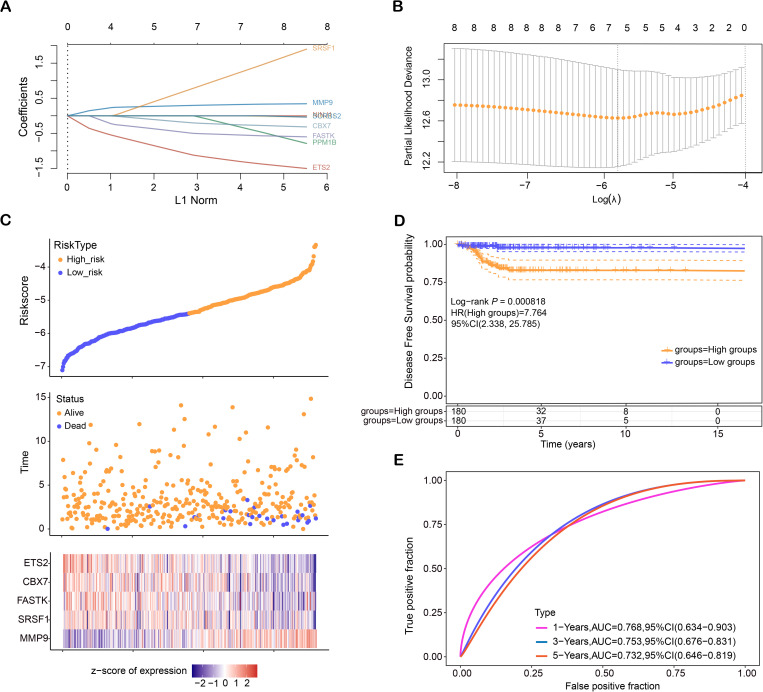
LASSO Regression and Risk Model Evaluation for Prognostic Gene Identification in THCA. **(A)** LASSO regression plot depicting gene selection across different lambda values, with optimal lambda highlighted. **(B)** The coefficient plot from LASSO regression indicates the eight prognostic genes selected based on their coefficients. **(C)** Risk score distribution of TCGA-THCA patients (Top). Blue represents the low risk, and pink represents the high risk. Scatter plot of patient survival status (Middle). Blue represents the dead, and orange represents the alive. Heatmap of 5 prognostic signature gene expression profiles from low-risk to high-risk patients (Bottom). **(D)** Kaplan-Meier survival curves comparing overall survival between high-risk and low-risk groups based on the risk model. **(E)** ROC curves and AUC values for the risk model at 1-year, 3-year, and 5-year intervals, with the highest AUC at one year. LASSO: Least Absolute Shrinkage and Selection Operator; AUC: Area Under the Curve; ROC: Receiver Operating Characteristic.

LASSO proportional hazards regression, combined with 10-fold cross-validation, was employed to detect potential prognostic genes. The optimal gene features were selected at lambda. min = 0.0048, resulting in the identification of eight candidate genes (Figs 2A, 2B). Risk scores were then determined for every patient, and based on the median cutoff point, patients were stratified into high-risk and low-risk groups. As shown in Fig 2C, a heatmap displays the survival outcomes of all patients and the expression patterns of the five characteristic genes (*ETS2*, *CBX7*, *FASTK*, *SRSF1*, *MMP9*). The Kaplan-Meier survival analysis demonstrates a significant difference in DFS between the high-risk and low-risk groups (**P* *= 0.000818). Patients in the high-risk group have a markedly worse DFS, with a hazard ratio (HR) of 7.764 (95% CI: 2.338–25.785), indicating they are approximately 7.8 times more likely to experience disease recurrence or progression compared to the low-risk group. The high-risk group shows a sharp decline in DFS probability within the first 5 years, while the low-risk group maintains a high DFS probability over time, highlighting the prognostic value of risk stratification (Fig 2D). The ROC curves that are time-dependent proved the prognostic accuracy of the risk model, with the highest AUC observed at one year (AUC = 0.768), reflecting strong predictive performance (Fig 2E).

### Prognostic value of ETS2 and clinical variables in THCA

**Fig 3:**
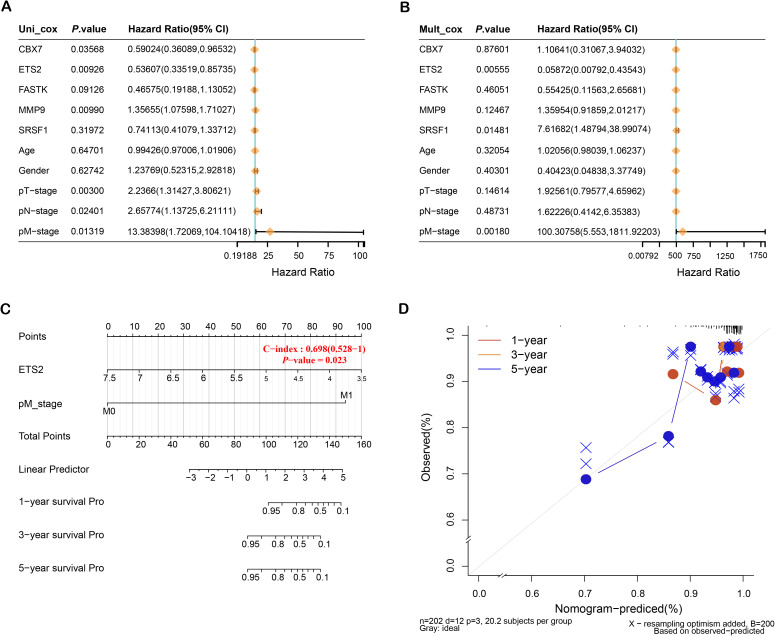
Cox regression analysis and nomogram for prognostic evaluation of THCA. **(A)** Univariate Cox regression analysis of prognostic signature genes and clinical variables, displaying hazard ratios (HR) and 95% confidence intervals (CI). **(B)** Multivariate Cox regression analysis assessing the prognostic value of prognostic signature genes along with clinical variables. **(C)** Nomogram for predicting 1-year, 3-year, and 5-year survival probabilities based on key prognostic factors, including *ETS2*. **(D)** Calibration plots comparing observed versus predicted survival probabilities for 1-year, 3-year, and 5-year survival.

Investigations of the five distinctive genes and clinical factors by univariate and multivariate Cox regression revealed that *ETS2* and pM stage are key prognostic factors (Figs 3A, 3B). Consequently, *ETS2* was found to be a critical gene in this study. The nomogram analysis and calibration curves for key prognostic variables further demonstrated that *ETS2* possesses excellent predictive capability for the survival of patients at 1, 3, and 5 years (Figs 3C, 3D). These results imply that *ETS2* could represent a valuable target for therapy and prognostic biomarkers in THCA.

### Differential expression and prognostic significance of ETS2 in THCA

**Fig 4:**
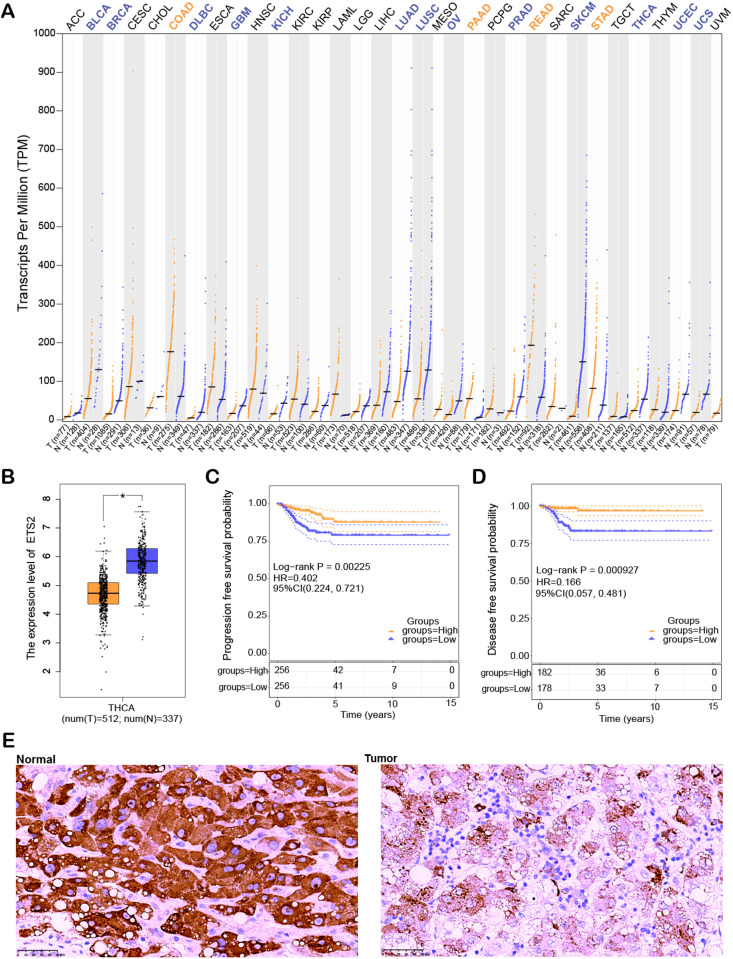
Expression and prognostic relevance of *ETS2* in THCA. **(A)** Pan-cancer analysis showing the expression levels of *ETS2* across various cancer types, including THCA, with data represented as transcripts per million (TPM). **(B)** Box plot of *ETS2* expression between THCA tumor tissue (T) and adjacent normal tissue **(N)**. **(C)** Kaplan-Meier survival curves for progression-free survival (PFS) stratified by *ETS2* expression levels. **(D)** Kaplan-Meier survival curves for disease-free survival (DFS) associated with *ETS2* expression levels. **(E)** Immunohistochemical analysis of *ETS2* expression in normal thyroid tissue compared to THCA tumor tissue. *P < 0.05.

Pan-cancer analysis utilizing the GEPIA2 database revealed significant variation in *ETS2* expression across multiple cancer types. Notably, *ETS2* was discovered to be downregulated in various cancers, such as THCA, bladder cancer (BLCA), diffuse large B-cell lymphoma (DLBC), glioblastoma multiforme (GBM), and breast cancer (BRCA), with the most pronounced downregulation observed in THCA (Figs 4A, 4B). revealed a substantial correlation between lower PFS and DFS and low *ETS2* expression (Figs 4C, 4D). Additionally, IHC analysis demonstrated that *ETS2* expression was markedly higher in normal tissue relative to THCA tumor tissue (Fig 4E). These outcomes suggest that *ETS2* expression level perhaps connected to a poor prognosis and that it may be important in the initiation and development of THCA.

### ETS2 overexpression inhibits THCA cell behavior

**Fig 5:**
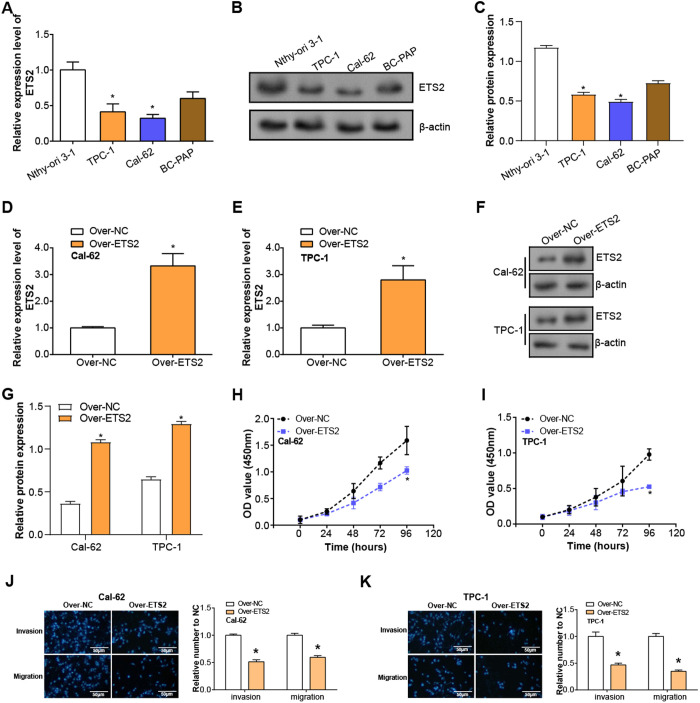
Effects of *ETS2* overexpression on THCA cell behavior. **(A)** qRT-PCR analysis of *ETS2* mRNA expression in normal thyroid epithelial cells (Nthy-ori 3-1) and THCA cell lines (Cal-62, TPC-1, BC-PAP). **(B-C)** WB analysis showing ETS2 protein expression in normal thyroid epithelial cells versus thyroid cancer cell lines. **(D-G)** Verification of *ETS2* overexpression in Cal-62 and TPC-1 cells through qRT-PCR and WB assays. (H and **I)** CCK-8 assay illustrating the proliferation rates of Cal-62 and TPC-1 cells post-*ETS2* overexpression. **(J and K)** Transwell assay quantifying invasion and migration capabilities of Cal-62 and TPC-1 cells following *ETS2* overexpression. Scale bar: 50 μm. qRT-PCR: Quantitative Reverse Transcription Polymerase Chain Reaction; WB: Western Blotting; CCK-8: Cell Counting Kit-8. *P < 0.05.

The expression of *ETS2* in several THCA cell lines (TPC-1, Cal-62, BC-PAP) was analyzed and compared with normal thyroid cells (Nthy-ori 3–1). qRT-PCR outcomes demonstrated that the mRNA expression of *ETS2* in both THCA cell lines (TPC-1, Cal-62) was significantly downregulated compared with normal thyroid cells (Fig 5A). WB analysis further verified the decreased protein level of ETS2 in these cancer cell lines (Fig 5B, 5C). qRT-PCR and WB verified the transfection efficiency of *ETS2* overexpression plasmid in TPC-1 and Cal-62cells and confirmed the high expression of *ETS2* (Figs 5D–5G). CCK-8 assay showed a significant decrease in cell proliferation after *ETS2* overexpression, suggesting that it has a suppressive impact on the proliferation of THCA cells (Fig 5H, 5I). In addition, Transwell experiments also found that after *ETS2* overexpression, the quantity of invasive and migrating cells was considerably decreased, suggesting that *ETS2* markedly reduced the migration and invasion ability of THCA cells (Figs 5J, 5K), further supporting the function of *ETS2* overexpression in inhibiting the proliferation, invasion, and migration of THCA cells.

### ETS2 overexpression promotes apoptosis in THCA cells

**Fig 6:**
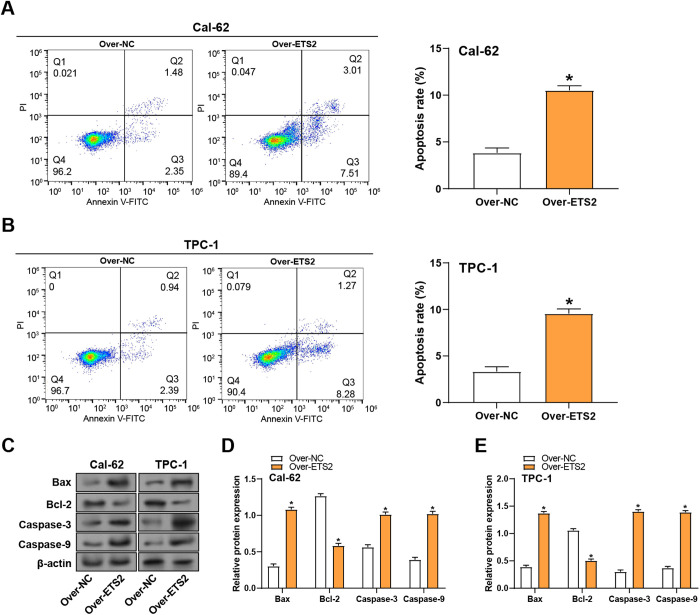
Apoptotic effects of *ETS2* overexpression in THCA cells. **(A and B)** Flow cytometry analysis showing apoptosis rates in Cal-62 and TPC-1 cells with *ETS2* overexpression. **(C)** WB analysis of apoptosis-related proteins (Bax, Bcl-2, Caspase-3, Caspase-9) in THCA cells with *ETS2* overexpression. **(D-E)** Bar graph quantifying levels of pro-apoptotic (Bax, Caspase-3, Caspase-9) and anti-apoptotic (Bcl-2) proteins with *ETS2* overexpression. WB: Western Blotting. *P < 0.05.

Flow cytometry was utilized to evaluate the impact of *ETS2* overexpression on apoptosis in Cal-62 and TPC-1 cells. The outcomes revealed a notable rise in the quantity of Cal-62 and TPC-1 apoptotic cells following *ETS2* overexpression, indicating that *ETS2* enhances apoptosis in thyroid cancer cells (Figs 6A, 6B). WB analysis was conducted to assess the results of *ETS2* overexpression on apoptosis-related proteins, including Bax, Bcl-2, Caspase-9, and Caspase-3. The outcomes revealed that *ETS2* overexpression led to increased expression of Caspase-9, Bax, and Caspase-3, while Bcl-2 expression was decreased in Cal-62 and TPC-1 cells (Figs 6C–6E). These findings suggest that *ETS2* overexpression promotes apoptosis in THCA cells by modulating the expression of key apoptotic regulators.

### ETS2 overexpression regulates EMT marker expression in THCA cells

**Fig 7:**
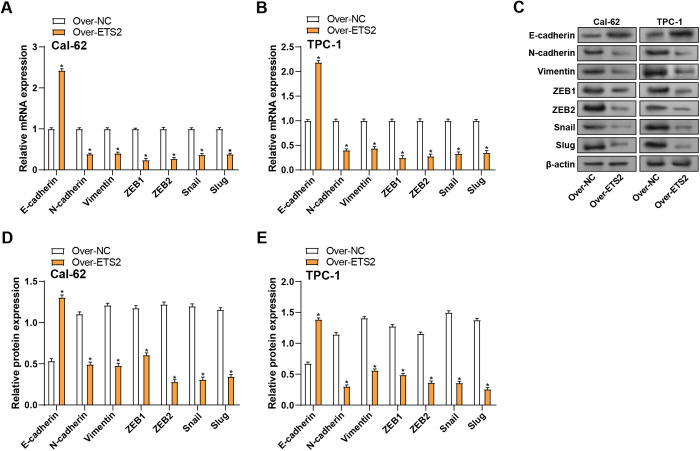
Effect of *ETS2* overexpression on the expression of EMT markers in THCA cells. **(A-B)** qRT-PCR analysis of EMT-related gene mRNA levels (*E-cadherin*, *N-cadherin*, *Vimentin*, *ZEB1*, *ZEB2*, *Snail*, *Slug*) in THCA cells with *ETS2* overexpression. **(C)** WB analysis confirms the effects of *ETS2* overexpression at the protein level. **(D-E)** Quantification of EMT protein expression levels with *ETS2* overexpression. qRT-PCR: Quantitative Reverse Transcription Polymerase Chain Reaction; WB: Western Blotting; EMT: Epithelial-Mesenchymal Transition. *P < 0.05.

The EMT process enables cancer cells to acquire enhanced migratory and invasive capabilities. This study investigated the impact of *ETS2* overexpression on the levels of proteins linked to EMT in THCA cells. qRT-PCR findings showed that *ETS2* overexpression suppressed the mRNA expression of *N-cadherin*, *Vimentin*, *ZEB1*, *ZEB2*, *Slug*, and *Snail* and enhanced the mRNA expression of *E-cadherin* in comparison to the control group (Figs 7A, 7B). WB analysis corroborated these findings at the protein level (Figs 7C–7E). These results demonstrate that *ETS2* overexpression can reverse the EMT process in THCA cells, suggesting a possible involvement in preventing progression and metastasis.

### Validation of the interaction between ETS2 and ZMYND11 in THCA cells

**Fig 8:**
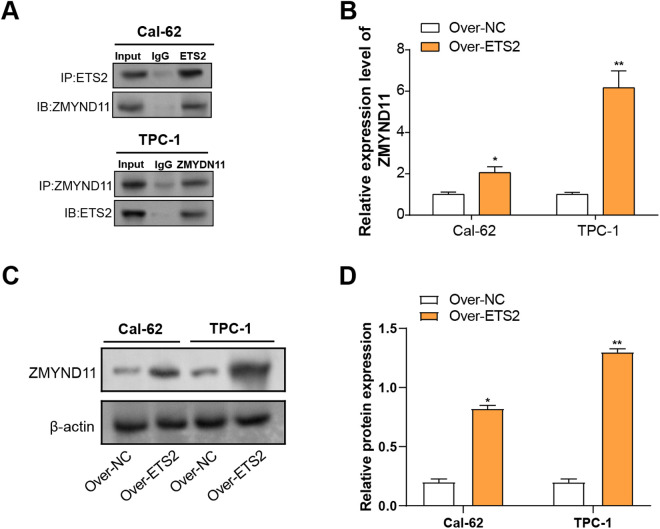
Interaction between *ETS2* and *ZMYND11* in THCA cells. **(A)** Co-immunoprecipitation results confirm the interaction between ETS2 and ZMYND11 in Cal-62 and TPC-1 cells. IP: Immunoprecipitation, IN: input. **(B)** qRT-PCR analysis showing the impact of *ETS2* overexpression on *ZMYND11* expression in Cal-62 and TPC-1 cells. **(C)** WB analysis of ZMYND11 expression levels in cells with ETS2 overexpression. **(D)** Bar graph illustrating changes in ZMYND11 expression due to ETS2 overexpression. qRT-PCR: Quantitative Reverse Transcription Polymerase Chain Reaction; WB: Western Blotting. *P < 0.05, **P < 0.01.

To learn more about the connection between *ETS2* and *ZMYND11*, Co-IP and expression analysis were performed. Co-IP results demonstrated that ZMYND11 interacts with ETS2 in the THCA cell lines (Fig 8A). Subsequent qRT-PCR analysis revealed that *ETS2* overexpression significantly increased *ZMYND11* mRNA levels in both cell lines, with a more pronounced effect observed in TPC-1 cells (Fig 8B). In line with the mRNA results, WB analysis verified a noteworthy rise in ZMYND11 protein levels following ETS2 overexpression in THCA cell lines (Figs 8C, 8D). These findings suggest that *ETS2* positively regulates *ZMYND11* expression in THCA cells.

### ZMYND11 knockdown attenuates the inhibitory effect of ETS2 overexpression on THCA cell behavior

**Fig 9:**
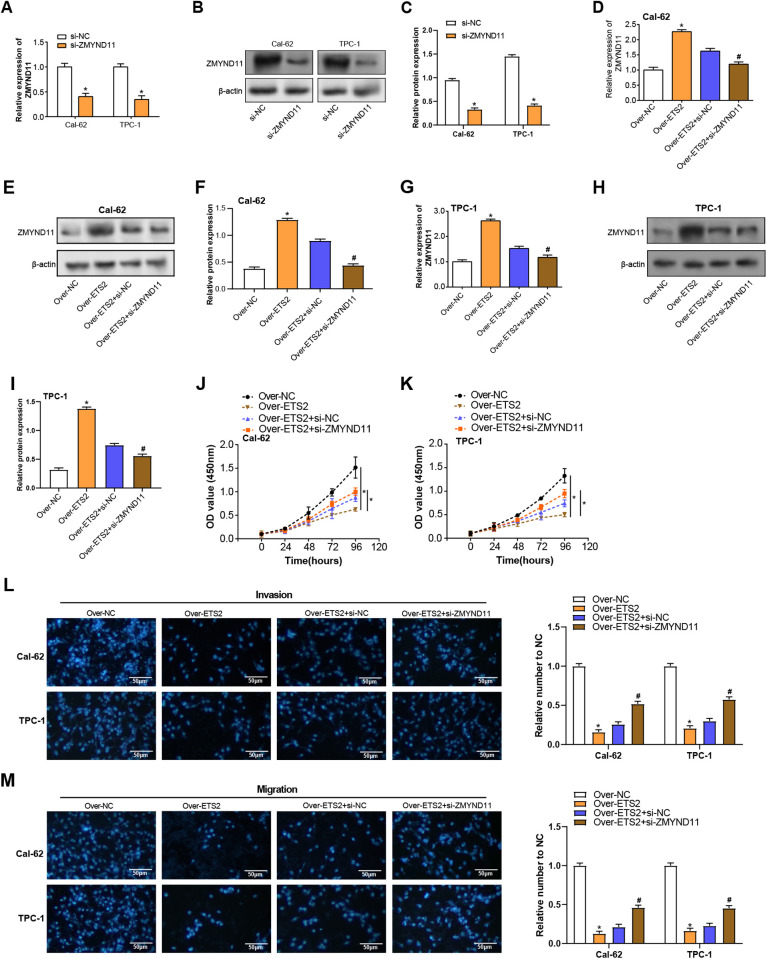
*ZMYND11* knockdown reverses the effects of *ETS2* overexpression on cell proliferation, migration, and invasion in THCA cells. **(A)** qRT-PCR analysis of *ZMYND11* knockdown efficiency in Cal-62 and TPC-1 cells. **(B)** WB analysis showing ZMYND11 protein expression levels post-knockdown. **(C)** Bar graph illustrating a reduction in ZMYND11 expression following knockdown. **(D)** qRT-PCR results assessing *ZMYND11* levels in Cal-62 cell with *ETS2* overexpression and *ZMYND11* knockdown. **(E-F)** WB analysis of ZMYND11 expression in Cal-62 cell with combined ETS2 overexpression and ZMYND11 knockdown. **(G)** qRT-PCR results assessing *ZMYND11* levels in TPC-1 cell with *ETS2* overexpression and *ZMYND11* knockdown. **(H-I)** WB analysis of ZMYND11 expression in TPC-1 cell with combined ETS2 overexpression and ZMYND11 knockdown. **(J-K)** CCK-8 assay depicting cell proliferation in Cal-62 and TPC-1 cells with *ETS2* overexpression and *ZMYND11* knockdown. **(L-M)** Transwell assay results for cell invasion and migration in Cal-62 and TPC-1 cells with *ETS2* overexpression and *ZMYND11* knockdown. Scale bar: 50 μm. qRT-PCR: Quantitative Reverse Transcription Polymerase Chain Reaction; WB: Western Blotting; CCK-8: Cell Counting Kit-8. *P < 0.05, ^#^*P* < 0.05.

To investigate the functional relationship between *ETS2* and *ZMYND11* in THCA cells, *ZMYND11* knockdown was performed using siRNA in Cal-62 and TPC-1 cells. qRT-PCR and WB analysis confirmed that *ZMYND11* mRNA and protein levels were greatly decreased after siRNA transfection in comparison to the control (Fig 9A–9C). Subsequent knockdown of *ZMYND11* in *ETS2*-overexpressing cells caused *ZMYND11* expression to significantly decline at the levels of proteins and Mrna (Fig 9D–9F). Functionally, the knockdown of *ZMYND11* partially reversed the restraint of *ETS2* overexpression on cell growth, as confirmed by the CCK-8 assay in both TPC-1 and Cal-62 cells (Fig 9J, 9K). Furthermore, Transwell assays indicated that the enhanced invasion and migration abilities observed in *ETS2*-overexpressing cells were significantly attenuated after *ZMYND11* knockdown (Figs 9L, 9M). These results suggest that *ZMYND11* mediates the way that *ETS2* affects THCA cell invasion, migration, and proliferation, highlighting the functional interaction between these two genes.

### ZMYND11 knockdown reverses ETS2-mediated apoptosis in THCA cells

**Fig 10:**
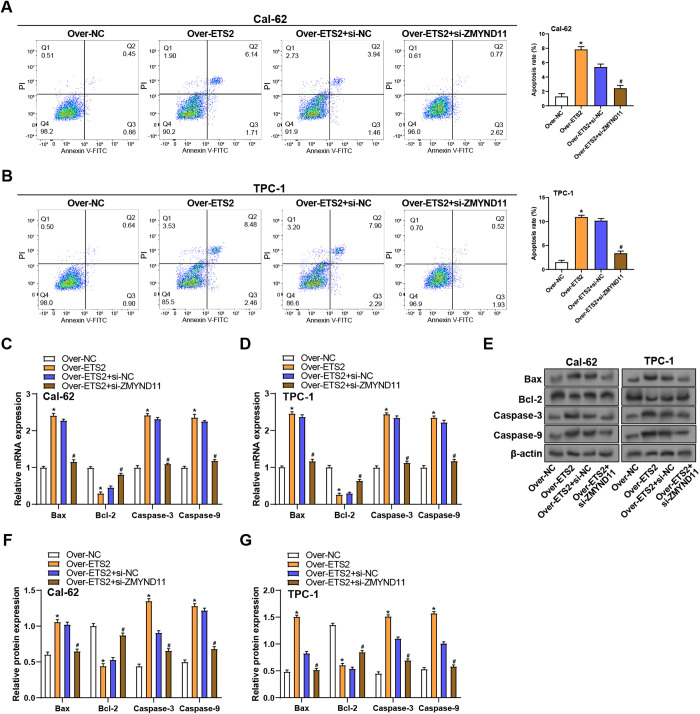
*ZMYND11* knockdown reverses *ETS2*-induced apoptosis in THCA cells. **(A and B)** Flow cytometry analysis showing apoptotic rates in Cal-62 and TPC-1 cells with *ETS2* overexpression and/or *ZMYND11* knockdown. **(C** and **D)** qRT-PCR analysis of apoptosis-related genes (*Bax*, *Bcl-2*, *Caspase-3*, *Caspase-9*) under *ETS2* overexpression and *ZMYND11* knockdown conditions. **(E)** WB analysis of apoptosis-related proteins (Bax, Bcl-2, Caspase-3, Caspase-9) under *ETS2* overexpression and *ZMYND11* knockdown conditions. **(F and G)** Bar graph quantifying changes in apoptosis-related protein levels. qRT-PCR: Quantitative Reverse Transcription Polymerase Chain Reaction; WB: Western Blotting. *P < 0.05, ^#^*P* < 0.05.

To discover the role of *ZMYND11* in *ETS2*-induced apoptosis in THCA cells, flow cytometry and expression analysis of apoptosis-related genes were performed. Flow cytometry with Annexin V/PI staining demonstrated that *ETS2* overexpression notably elevated apoptosis in TPC-1 and Cal-62 cells relative to the control group. However, *ZMYND11* knockdown in *ETS2*-overexpressing cells markedly reduced the percentage of apoptotic cells (Figs 10A, 10B). Gene expression analysis by qRT-PCR showed that *ETS2* overexpression led to a significant rise in pro-apoptotic genes and a decrease in the anti-apoptotic gene in Cal-62 and TPC-1 cells. Knockdown of *ZMYND11* reversed these changes, restored *Bcl-2* levels, and reduced the levels of pro-apoptotic genes (Figs 10C, 10D). WB analysis further confirmed these findings at the protein level, indicating that in *ETS2*-overexpressing cells, *ZMYND11* knockdown decreased Caspase-9, Caspase-3, and Bax levels while raising Bcl-2 protein expression (Figs 10E, 10F, 10G). These findings imply that ZMYND11 is crucial for mediating the ETS2-induced apoptosis that occurs in THCA cells.

### ZMYND11 knockdown reverses ETS2-induced EMT marker expression in THCA cells

**Fig 11:**
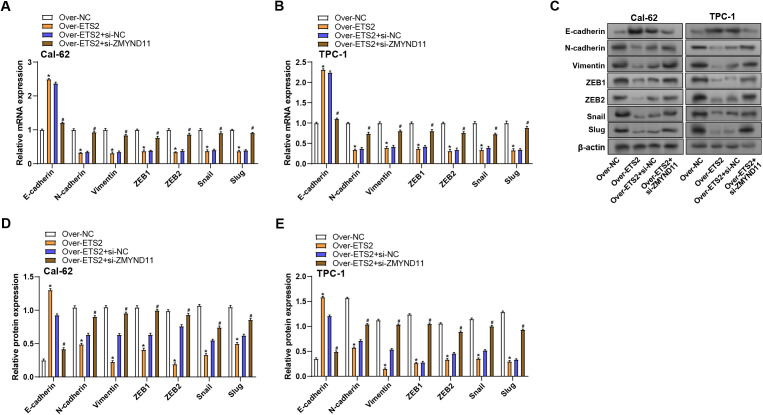
*ZMYND11* knockdown reverses *ETS2*-induced changes in EMT marker expression in THCA cells. **(A)** qRT-PCR analysis of EMT marker (*E-cadherin*, *N-cadherin*, *Vimentin*, *ZEB1*, *ZEB2*, *Snail*, *Slug*) in Cal-62 cells after *ETS2* overexpression and/or *ZMYND11* knockdown. **(B)** Bar graph showing the changes in the mRNA levels of EMT markers in TPC-1 cells after *ETS2* overexpression and/or *ZMYND11* knockdown. **(C)** WB analysis of EMT proteins (E-cadherin, N-cadherin, Vimentin, ZEB1, ZEB2, Snail, Slug) following *ETS2* overexpression and/or *ZMYND11* knockdown. **(D and E)** Quantification of EMT protein expression levels with *ETS2* overexpression and/or *ZMYND11* knockdown. EMT: Epithelial-Mesenchymal Transition; qRT-PCR: Quantitative Reverse Transcription Polymerase Chain Reaction; WB: Western Blotting. *P < 0.05, ^#^*P* < 0.05.

We investigated the impacts of *ETS2* overexpression and/or *ZMYND11* knockdown on the expression levels of EMT-related proteins to learn more about whether *ZMYND11* and *ETS2* affect the EMT process in THCA cells. qRT-PCR results demonstrated that *ETS2* overexpression markedly elevated the levels of *E-cadherin* while suppressing the levels of *Vimentin*, *N-cadherin*, *ZEB1*, *ZEB2*, *Snail*, and *Slug* in contrast to the group under control. On the other hand, *ETS2* overexpression and *ZMYND11* knockdown caused a decline in *E-cadherin* expression and a significant increase in *Vimentin*, *N-cadherin*, *ZEB1*, *ZEB2*, *Snail*, and *Slug* expression (Figs 11A, 11B). These results were further supported by WB analysis, which validated the observed changes at the protein level (Figs 11C–11E). Overall, these findings suggest that *ETS2* is essential for controlling the EMT process, and its effects may be mediated through modulation of *ZMYND11* expression.

### ZMYND11 knockdown regulates ETS2-mediated regulation of mTOR pathway-related genes

**Fig 12:**
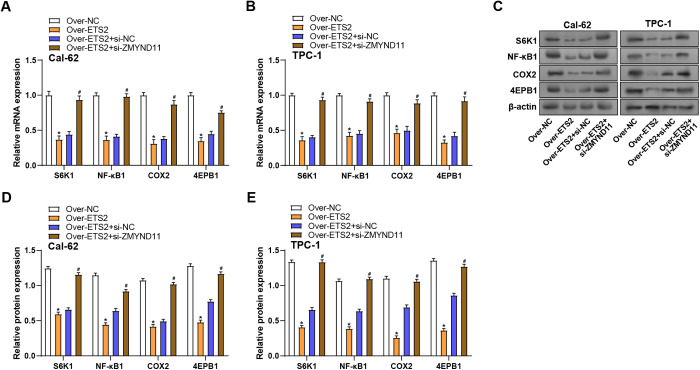
*ZMYND11* knockdown reverses *ETS2*-mediated downregulation of mTOR signaling pathway-related genes in THCA cells. **(A** and **B)** qRT-PCR analysis of mTOR pathway-related genes (*S6K1*, *NF-κB1*, *COX2*, *4EBP1*) in Cal-62 and TPC-1 cells with *ETS2* overexpression and/or *ZMYND11* knockdown. **(C)** WB analysis of mTOR signaling pathway proteins (S6K1, NF-κB1, COX2, 4EBP1) in Cal-62 and TPC-1 cells with *ETS2* overexpression and/or *ZMYND11* knockdown. **(D-E)** Bar graph quantifying mTOR pathway protein levels with *ETS2* overexpression and/or *ZMYND11* knockdown. qRT-PCR: Quantitative Reverse Transcription Polymerase Chain Reaction; WB: Western Blotting. *P < 0.05, ^#^*P* < 0.05.

Finally, to explore the effect of *ZMYND11* on *ETS2*-mediated regulation of genes linked to the mTOR pathway, we analyzed the expression levels of key signaling molecules in THCA cells. qRT-PCR analysis revealed that *ETS2* overexpression significantly decreased the mRNA expression in both cell lines. However, the knockdown of *ZMYND11* in *ETS2*-overexpressing cells reversed this effect, resulting in a notable rise in the levels of *S6K1*, *NF-κB1*, *COX2*, and *4EBP1* (Figs 12A, 12B). Similar to the mRNA results, this result was also confirmed at the protein level (Figs 12C–12E). These findings suggest that *ZMYND11* is essential for controlling *ETS2*-mediated regulation of the mTOR signaling cascade in THCA cells.

### *ETS2* overexpression suppresses tumor growth *in vivo*

**Fig 13:**
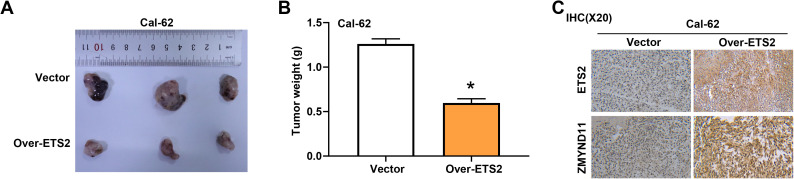
Overexpression of *ETS2* inhibits tumor growth *in vivo.* **(A)** Representative images of xenograft tumors formed by Cal-62 cells transfected with empty vector or *ETS2*-overexpressing plasmid (over-*ETS2*) in nude mice. **(B)** Quantification of tumor weight in the vector and over-*ETS2* groups. *P < 0.05. **(C)** IHC staining of ETS2 and ZMYND11 in xenograft tumor tissues. Magnification: 20x. IHC: Immunohistochemical.

To further validate the tumor-suppressive role of *ETS2* in thyroid cancer, we established a mouse xenograft model using Cal-62 cells overexpressing *ETS2* or a control vector. The results showed that, compared with the control group, tumors derived from the *ETS2* overexpression group exhibited a significantly reduced weight, indicating that *ETS2* inhibits tumor growth in vivo (Fig 13A, 13B). In addition, IHC staining was performed on xenograft tumor tissues to assess the expression of ETS2 and ZMYND11 (Fig 13C). The results revealed a marked increase in both ETS2 and ZMYND11 expression in the *ETS2* overexpression group compared to the vector group. These findings are consistent with our *in vitro* data and suggest that *ETS2* not only inhibits tumor growth but may also positively regulate *ZMYND11* expression in thyroid cancer tissues.

## Discussion

Previous investigations have identified the presence of senescent cells and cancer-associated fibroblasts (CAFs) in various thyroid cancer subtypes, including papillary, poorly differentiated, and anaplastic thyroid cancers [16]. These cells were found to coexist at the invasive edges of tumors, particularly in cases with BRAF mutations or BRAF-like molecular subtypes, correlating with local invasion [17]. In the present study, analysis of the TCGA-THCA dataset identified 61 DEGs associated with cellular senescence pathways, with *ETS2* emerging as a key prognostic gene. ETS2, known for its dual role in regulating cell proliferation and apoptosis, was markedly downregulated in THCA, and lower expression of *ETS2* was connected to poorer PFS and DFS. IHC analysis confirmed reduced ETS2 protein levels in THCA tissues in contrast to typical tissues, underscoring its capability to serve as a therapeutic target and biomarker. Additionally, previous research by Ren Y et al. highlighted distinct roles of *ETS1* and *ETS2* across cancers, with *ETS1* involved in oxidative stress regulation and ETS2 in transcriptional control [18]. Thus, further investigation into the function of *ETS2* in THCA could offer insightful information about therapy.

*ETS2*, a gene involved in cellular response and senescence, is a key factor in thyroid cancer progression [19]. A complicated relationship between *ETS2* and several signaling pathways has been identified by recent investigations. For instance, Yin X. et al. demonstrated that elevated exosomal miR-663b in bladder cancer targets *ETS2* inhibitors and promotes proliferation and EMT [20]. Similarly, Ichikawa MK. et al. highlighted the function of *ETS2* in senescence, enhancing the levels of *Snail* and *ZEB1/2* under TGF-β and Ras signaling [21]. EMT plays a crucial part in embryonic development, tissue regeneration, and cancer metastasis [22]. It is defined by the upregulation of mesenchymal markers like *Vimentin* and *N-cadherin* and the reduction of epithelial markers like *E-cadherin*. This process is regulated by transcription-related factors including *Slug*, *Snail*, *ZEB2*, and *ZEB1*. In THCA, EMT enhances invasiveness and metastasis, leading to a poor prognosis. Genes and pathways associated with EMT, including PI3K/AKT and MAPK/EMT, have been implicated in THCA progression [23]. Inhibition or reversal of EMT, for example by targeting the interaction of *HER2* with the MAPK/EMT pathway, could provide a potential therapeutic strategy. EMT also supports the maintenance of stem cells for cancer, increasing tumor invasiveness and treatment refractoriness [24]. Our results demonstrate that *ETS2* overexpression in THCA cells inhibits migration, proliferation, and invasion while enhancing apoptosis and regulating the levels of EMT-related markers. This highlights *ETS2* as a possible therapeutic objective for THCA.

The results of this investigation underscore the significant regulatory roles of *ETS2* and *ZMYND11* in THCA and their impact on tumor progression. *ZMYND11*, characterized by its MYND domain, is crucial in protein-protein interactions and transcriptional regulation. In cancer, *ZMYND11* modulates gene expression related to the cell cycle, differentiation, and apoptosis, influencing tumor growth [25]. For instance, Zhang Z. et al. identified that elevated microRNA-196b in ovarian cancer promotes cell proliferation, invasion, and migration by targeting *ZMYND11*, suggesting that its function can be modulated by specific microRNAs, with abnormal expression contributing to malignancy [26]. Additionally, Meng X. et al. demonstrated that USP53 interacts with *ZMYND11* to enhance its deubiquitination and stability, inhibiting breast cancer cell proliferation and inducing apoptosis [27]. Recent studies also suggest that ETS1, another member of the ETS transcription factor family, may crosstalk with ETS2 in regulating ZMYND11 expression and its downstream effects [13]. ETS1 has been implicated in various cancers, including lung and breast cancer, where it cooperates with other transcription factors like ETS2 to influence gene expression related to cell invasion and metastasis [28]. The potential interaction between ETS1 and ZMYND11 in the context of THCA could provide a more complex regulatory mechanism underlying tumor progression. Our study confirms the direct interaction between *ETS2* and *ZMYND11* in THCA cells through Co-IP assay, showing that *ZMYND11* knockdown can counteract the impacts of overexpressing *ETS2* on invasion, proliferation, migration, apoptosis, and EMT. These findings emphasize the vital function of *ETS2* in regulating these processes, mediated through its interaction with *ZMYND11*. Understanding the *ETS2*-*ZMYND11* axis offers deeper insights into their collaborative role in thyroid cancer and suggests potential therapeutic targets for THCA management.

The mTOR pathway regulates essential cellular functions including proliferation, growth, metabolism, and survival, a crucial signaling network [29]. mTOR, a serine/threonine kinase, integrates environmental signals, including nutrients, oxygen, and energy status, to regulate cell behavior [30]. Earlier investigations have highlighted the function of mTOR in THCA. For example, Derwich A. et al. reviewed the expression of proteins and genes linked to mTOR in papillary thyroid carcinoma (PTC) and their association with disease risk and clinical outcomes [31]. Similarly, Lv J. et al. demonstrated that M2 tumor-associated macrophages (TAMs) promote the invasiveness and stemness of anaplastic thyroid carcinoma (ATC) cells by secreting IGF-1 and IGF-2, thereby activating the IR-A/IGF1R-mediated PI3K/AKT/mTOR signaling pathway [32]. mTORC1, a key part of the mTOR pathway, promotes protein synthesis by activating S6K1 and inhibiting eukaryotic translation initiation factor 4E-BP1 [33]. Additionally, the mTOR pathway can modulate NF-κB1 activity and influence COX2 expression, contributing to inflammation and cancer progression [34]. Our research examined the functions of *ETS2* and *ZMYND11* in modulating the mTOR signaling pathway in THCA cells. Our findings indicate that *ETS2* overexpression significantly reduces the expression levels of S6K1, NF-κB1, COX2, and 4E-BP1. However, when *ETS2* overexpression is combined with *ZMYND11* knockdown, the expression levels of these mTOR pathway components are restored. This suggests that the regulatory impact of *ETS2* on the mTOR pathway is mediated through its interaction with *ZMYND11*. These results highlight the complex interplay between *ETS2*, *ZMYND11*, and the mTOR signaling pathway in THCA cells, providing novel perspectives into potential pathways of THCA development and therapeutic targets.

In our study, while we have made significant progress in understanding the roles of *ETS2* and *ZMYND11* in thyroid cancer, we must acknowledge that our research has certain limitations. Firstly, a notable limitation of our study is the absence of in vivo validation using thyroid cancer animal models. Secondly, our investigation into the specific mechanisms by which ETS2 and ZMYND11 regulate the mTOR signaling pathway, while suggestive, lacks depth. The precise molecular interactions and downstream effects require further elucidation. Thirdly, our analysis of the correlation between ETS2 and ZMYND11 expression and patient prognosis, although indicative, is limited by a relatively small sample size. This restricts the generalizability of our conclusions. Lastly, our study did not yield breakthrough findings in terms of novel mechanisms. The field is in need of innovative discoveries that can significantly advance our understanding of THCA pathogenesis. In summary, while our study provides a comprehensive analysis of ETS2 and ZMYND11 in THCA, it is essential to address these limitations through future research that incorporates in vivo models, advanced molecular techniques, larger sample sizes, and a focus on uncovering novel mechanisms. This will lead to a more profound understanding of THCA and potentially identify new therapeutic strategies.

## Conclusion

This study shows that the cellular senescence-related gene *ETS2* has a major function in controlling cell proliferation, apoptosis, and EMT in THCA. Overexpression of *ETS2* inhibits the growth, invasion, and migration of THCA cells while promoting apoptosis. The interaction between *ETS2* and *ZMYND11*, and knockdown of *ZMYND11* reverses these effects, restoring key components of the mTOR pathway and EMT markers. These results emphasize the significance of the *ETS2*-*ZMYND11* axis in cellular senescence and thyroid cancer progression, underscoring its possible use as a THCA treatment target.

## Supporting information

Supplementary File 1 Unprocessed original Western blot images for Figures 5B–12C. (PDF)

Supplementary File 2 Unprocessed original Co-IP blots for Figure 8A. (RAR)

Supplementary File 3 Unprocessed raw data of Transwell invasion and migration assays in Figures 9L and 9M. (RAR)

Supplementary File 4 Raw data files for apoptosis analysis are presented in Figures 10A and 10B. (RAR)
