# Supplementary material for: ETS2 targets ZMYND11 to inhibit thyroid cancer progression via the mTOR signaling pathway
Source: PLoS One. 2025 Sep 12;20(9):e0328881. doi: 10.1371/journal.pone.0328881 (PMC12431355; doi:10.1371/journal.pone.0328881)
Supplement: Supplementary File 1 — (PDF) [file pone.0328881.s001.pdf]

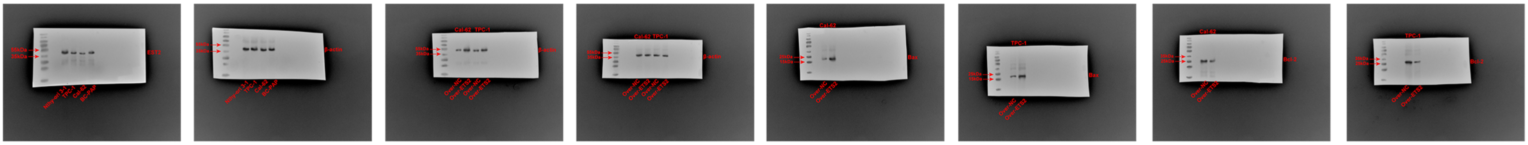

Figure 5B_EST2 | Figure 5B_β-actin | Figure 5F_ETS2 | Figure 5F_β-actin | Figure 6C_Bax_Cal-62 | Figure 6C_Bax_TPC-1 | Figure 6C_Bcl-2_Cal-62 | Figure 6C_Bcl-2_TPC-1

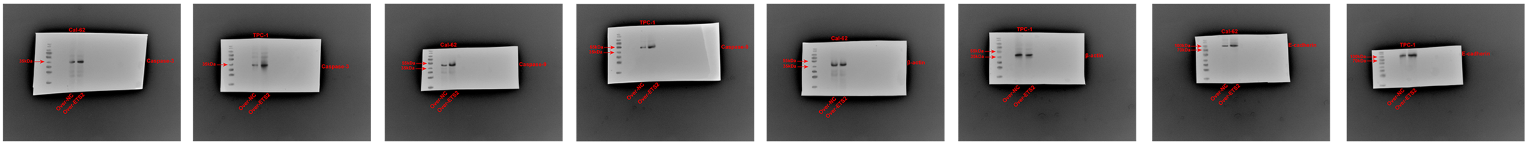

Figure 6C_Caspase-3_Cal-62 | Figure 6C_Caspase-3_TPC-1 | Figure 6C_Caspase-9_Cal-62 | Figure 6C_Caspase-9_TPC-1 | Figure 6C_β-actin_Cal-62 | Figure 6C_β-actin_TPC-1 | Figure 7C_E-cadherin_Cal-62 | Figure 7C_E-cadherin_TPC-1

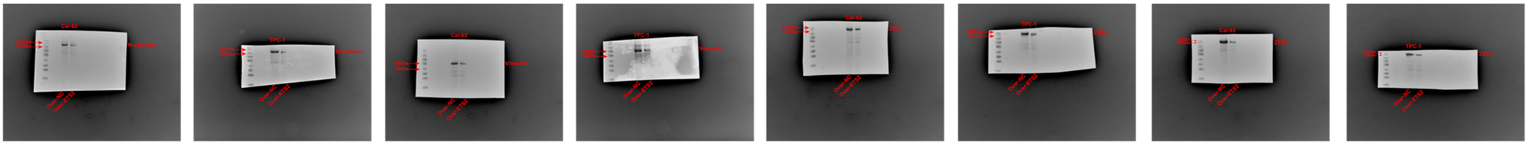

Figure 7C_N-cadherin_Cal-62 | Figure 7C_N-cadherin_TPC-1 | Figure 7C_Vimentin_Cal-62 | Figure 7C_Vimentin_TPC-1 | Figure 7C_ZEB1_Cal-62 | Figure 7C_ZEB1_TPC-1 | Figure 7C_ZEB2_Cal-62 | Figure 7C_ZEB2_TPC-1

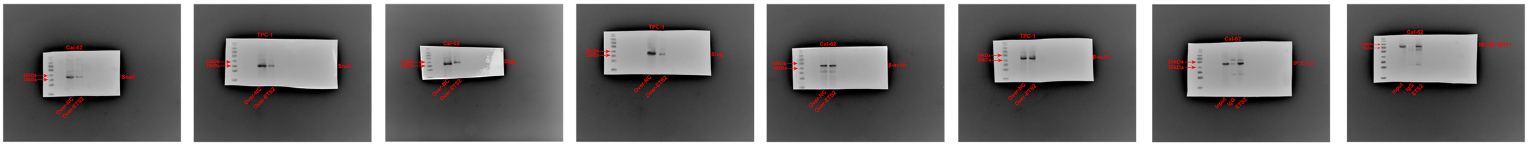

Figure 7C_Snail_Cal-62 | Figure 7C_Snail_TPC-1 | Figure 7C_Slug_Cal-62 | Figure 7C_Slug_TPC-1 | Figure 7C_β-actin_Cal-62 | Figure 7C_β-actin_TPC-1 | Figure 8A_IP_ETS2_Cal-62 | Figure 8A_IB_ZMYND11_Cal-62

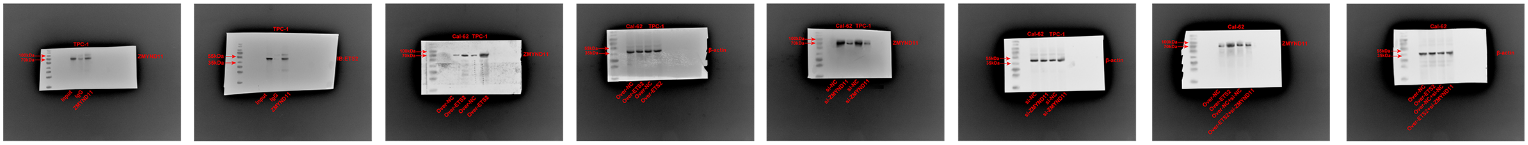

Figure 8A_IP_ZMYND11_TPC-1 | Figure 8A_IB_ETS2_TPC-1 | Figure 8C_ZMYND11 | Figure 8C_β-actin | Figure 9B_ZMYND11 | Figure 9B_β-actin | Figure 9E_ZMYND11 | Figure 9E_β-actin

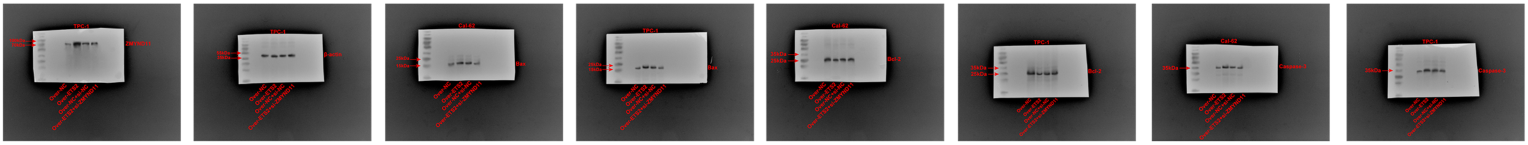

Figure 9H_ZMYND11 | Figure 9H_β-actin | Figure 10E_Bax_Cal-62 | Figure 10E_Bax_TPC-1 | Figure 10E_Bcl-2_Cal-62 | Figure 10E_Bcl-2_TPC-1 | Figure 10E_Caspase-3_Cal-62 | Figure 10E_Caspase-3_TPC-1

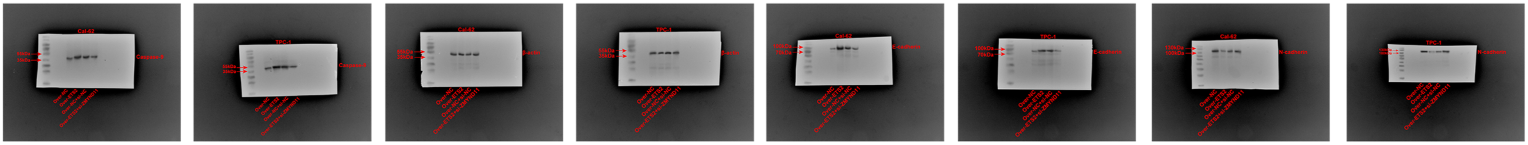

Figure 10E_Caspase-9_Cal-62 | Figure 10E_Caspase-9_TPC-1 | Figure 10E_β-actin_Cal-62 | Figure 10E_β-actin_TPC-1 | Figure 11C_E-cadherin_Cal-62 | Figure 11C_E-cadherin_TPC-1 | Figure 11C_N-cadherin_Cal-62 | Figure 11C_N-cadherin_TPC-1

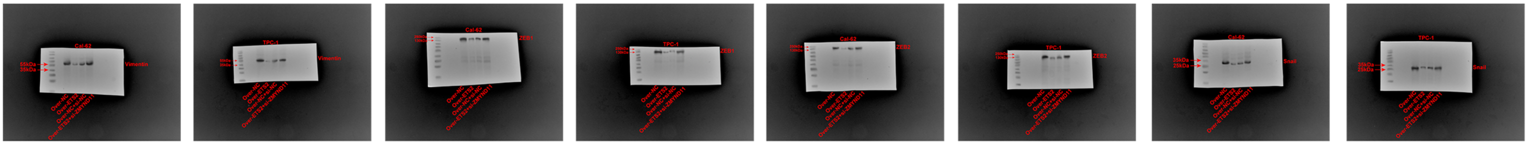

Figure 11C_Vimentin_Cal-62 | Figure 11C_Vimentin_TPC-1 | Figure 11C_ZEB1_Cal-62 | Figure 11C_ZEB1_TPC-1 | Figure 11C_ZEB2_Cal-62 | Figure 11C_ZEB2_TPC-1 | Figure 11C_Snail_Cal-62 | Figure 11C_Snail_TPC-1

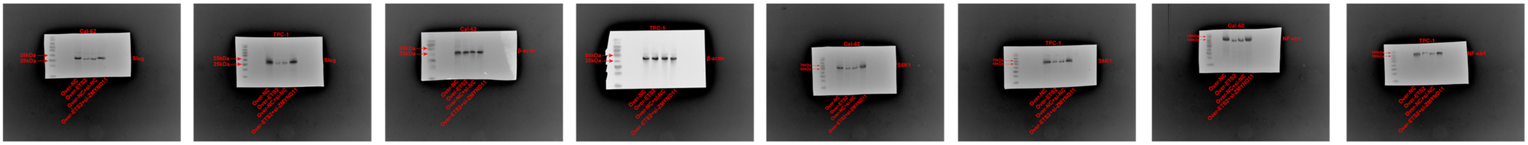

Figure 11C_Slug_Cal-62 | Figure 11C_Slug_TPC-1 | Figure 11C_β-actin_Cal-62 | Figure 11C_β-actin_TPC-1 | Figure 12C_S6K1_Cal-62 | Figure 12C_S6K1_TPC-1 | Figure 12C_NF-κB1_Cal-62 | Figure 12C_NF-κB1_TPC-1

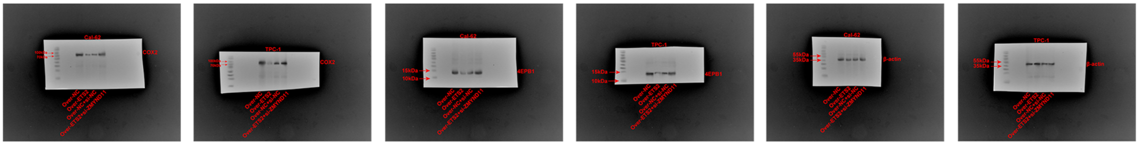

Figure 12C_COX2_Cal-62 | Figure 12C_COX2_TPC-1 | Figure 12C_4EPB1_Cal-62 | Figure 12C_4EPB1_TPC-1 | Figure 12C_β-actin_Cal-62 | Figure 12C_β-actin_TPC-1
